# Hydrofluoric Acid in Dentistry: An Investigation of Isolation and Neutralizing Agents and Precipitates on IPS e.max CAD

**DOI:** 10.3290/j.jad.b5883893

**Published:** 2024-12-17

**Authors:** Lisa Türp, Lucas Nehrke, Philipp Schadte, Leonard Siebert, Matthias Kern

**Affiliations:** a Assistant Professor, Department of Prosthodontics, Propaedeutics and Dental Materials, School of Dentistry, Christian-Albrechts University, Arnold-Heller-Strasse 3, House B, 24105 Kiel, Germany. Idea, experimental design, performed the experiments, wrote the manuscript.; b Assistant Professor, Department of Prosthodontics, Propaedeutics and Dental Materials, School of Dentistry, Christian-Albrechts University, Arnold-Heller-Strasse 3, House B, 24105 Kiel, Germany. Idea, experimental design, performed the experiments, editing.; c Academic Staff, Department of Material Science, Faculty of Engineering, Kiel University, Kaiserstr. 2, 24143 Kiel, Germany. Performed SEM/EDX measurements, editing.; d Academic Staff, Department of Material Science, Faculty of Engineering, Kiel University, Kaiserstr. 2, 24143 Kiel, Germany. Performed SEM/EDX measurements, editing.; e Chair, Department of Prosthodontics, Propaedeutics and Dental Materials, School of Dentistry, Christian-Albrechts University, Arnold-Heller-Strasse 3, House B, 24105 Kiel, Germany.

**Keywords:** acid etching, glass-ceramic repair, hydrofluoric acid, intraoral repair, isolation agent, lithium disilicate ceramic, neutralization, neutralizing agent

## Abstract

**Purpose:**

The purpose of this laboratory study was to evaluate common materials for isolation and neutralizing agents for hydrofluoric acid (HF). Additionally, surfaces of lithium disilicate ceramic were examined for precipitates after the etching and neutralizing process.

**Materials and Methods:**

The HF permeability of the following isolation agents (n=8) was investigated by positioning them over pH indicator paper under airtight conditions and applying 9% HF: latex rubber dam; elastic plastomer rubber dam; nitrile gloves; latex gloves; liquid rubber dam; Teflon; AZ strip. Four neutralizing agents were tested (n = 8): calcium carbonate plus chlorhexidine gel; calcium hydroxide; calcium D-gluconate monohydrate plus chlorhexidine gel; IPS neutralizing powder plus water. Each agent was mixed with HF, according to a calculated ratio, followed by determining the pH value. Forty lithium disilicate ceramic specimens were divided into five groups (n=8), according to the etching and neutralizing protocol, and examined for precipitates by scanning electron microscopy (SEM) and energy-dispersive X-ray (EDX) analysis.

**Results:**

All isolation agents were impermeable to HF, except for Teflon. HF could be neutralized with neutralizing agents under laboratory conditions, with mean central pH values ranging from 6 to 11. Assessment with SEM showed precipitates on surfaces when neutralizing with calcium hydroxide only. EDX analysis confirmed residues of calcium fluoride among others.

**Conclusion:**

All tested isolation agents, except for Teflon, seem suitable for clinical use. When using calcium hydroxide for neutralizing, precipitates could remain on the surface of lithium disilicate ceramic.

Hydrofluoric acid (HF), the inorganic acid of elemental fluorine, is commonly used in dentistry in gel or liquid form and is usually released for extraoral and rarely for intraoral application.^25^ HF mainly etches the crystalline leucite of glass ceramics, leaving behind microscopic glassy crypts, which creates a micromechanical etching pattern.^16,36^ Therefore, HF is used for etching glass ceramics prior to adhesive luting and also for intraoral repair.^8,24,35^ The concentrations of HF are usually ranging between 5% and 15%.^25^ The acid concentration and duration of HF etching affect the mechanical properties and resin bond strength to glass ceramics.^1,7,12,15,32,38,42,43^ Alternatives for the use of HF have been proposed, but despite its toxicity, etching with HF is still the gold standard, and for lithium disilicate ceramic etching with 5% HF for 20 s is well established.^17–19,26,33,41^

The toxicity of HF is based on fluoride ions that are strong scavengers of calcium and magnesium cations, forming insoluble salt, penetrating quickly through all layers of the epidermis, dermis, and deeper subcutaneous tissues causing severe destruction, necrosis, and injury to the bone by decalcification.^25,39^ The eye is also highly susceptible to HF liquid or vapor exposures, which may result in permanent eye damage.^20,27^ In case of inhalation or ingestion of HF, severe respiratory damage and systemic toxicity are of concern.^3,6,21,25^ A decrease in serum calcium (hypocalcemia) and other metabolic changes, may result in a fatal outcome if not recognized and treated.^34^ HF damage is related to the duration of exposure and the concentration of the acid. The majority of patients are burned at 1–3% concentration.^13,30,31^

Higher concentrations (>20%) of HF result in immediate visible burn and pain, whereas symptoms of erythema and pain after contamination with lower concentrations of HF (<20%) may be delayed up to 24 h. If untreated, it could also progress through the same sequence as the high-concentration burns.^21^ HF burns could be a result of the dehydrating effect of this acid and the low pH value. Skin contact with HF, even with a dilute solution (0.1%), can cause painful second- and third-degree burns that heal very slowly.^4^ In case of HF exposure, immediate skin surface irrigation with tap water should be initiated to remove HF from the skin and prevent rapid penetration by the extremely lipophilic acid before proceeding to the emergency department. After irrigation, the aim is to chemically sequester the fluoride ion and to prevent deep tissue destruction.^21^ As calcium ion decreases the toxic effects of sodium fluoride in tissue, a gel, solution, or injection of calcium gluconate is widely used for first aid and primary treatment.^14,21,40^

The primary goal of care is to avoid any HF exposure to skin, eyes, or inhalation or ingestion during dental treatment. In addition to gloves and safety glasses, a rubber dam and other isolation agents that serve as acid barriers seem mandatory when applying HF intraorally. For intraoral use of HF, a rubber dam, but also a liquid rubber dam, a neutralizing agent, or Teflon alone is recommended to protect the surrounding area that is not to be etched.^17,37^ But it is unclear whether common dental isolation agents are impermeable to HF.

As the use of glass-ceramic restorations is increasing^9^ and chippings have been reported as clinical problems,^22^ intraoral repair of fractured glass-ceramic restorations remains necessary. For intraoral use, a 9% HF gel is available.^37^ As precipitates are formed after HF etching that might remain on the glass-ceramic surface, making resin bonding more challenging, neutralizing agents and cleaning methods after etching have been suggested.^1,2,5,9,28^ However, there is no consensus on the influence of these procedures on the resin bond strength to ceramics; in particular, there is a lack of information on the neutralization process on lithium disilicate ceramic surfaces, precipitates, and their influence on the resin bond strength. Using a neutralization agent, a partially soluble powder of sodium carbonate and calcium carbonate, has been reported to result in a lower bond strength to feldspar ceramic than for groups that were not submitted to neutralization after etching.^2,5,28^ The presence of residual products of acid neutralization was assumed to be responsible for the reduced bonding potential.^2^ Another study used different neutralization agents on feldspar ceramic and observed that their use did not negatively affect the resin bond strength, concluding that these neutralizing agents should be considered as an alternative to reduce the toxicity of HF.^29^ Currently, no approved neutralizing agent for intraoral use is on the market. With regard to the environment and the safety of patients and clinicians, neutralizing and eliminating the fluoride ion of the HF is of interest.

The purpose of this laboratory study was to evaluate the HF permeability of various isolation agents and to investigate the effectiveness of four neutralizing agents, some of which may already exist in the dental clinic. Further, determining pH values and examining the surface of lithium disilicate ceramic for precipitates were of interest. The first hypothesis of this study was that all tested isolation agents would be impermeable to HF, and the second hypothesis was that all tested neutralization agents would be able to neutralize the HF.

## Materials and Methods

### Preliminary Tests

The pH value of a viscous buffered 9% HF for intraoral use (Porcelain Etch, Ultradent Products; South Jordan, UT, US) was determined by applying it directly to the pH indicator paper (Macherey-Nagel; Düren, Germany) using the manufacturer’s color scale chart.

**Fig 1:**
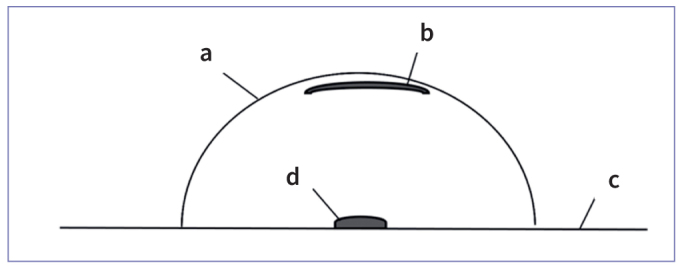
Schematic diagram of watch glass experiment: watch glass *(a)*; pH indicator paper *(b)*; carrier *(c)* with HF *(d)*.

A further preliminary test was conducted to investigate whether the HF evaporates. Therefore, a watch glass with a pH indicator paper attached to the top of the inner surface of the glass (with water) was placed above a carrier with intraoral HF (Fig 1). After an observation time of 60 s, changes in the pH indicator paper were determined.

Additionally, a pilot study to investigate isolation agents for HF permeability was performed as in the main study to determine a material that is impermeable to HF and suitable for the experimental set-up.

### Investigation of Isolation Agents

With regard to isolation agents that are used as a barrier to protect clinicians, patients, and the adjacent area that should not be etched during the intraoral application of HF, seven common materials were chosen (Table 1). The HF permeability of those isolation agents (n = 8) was investigated: latex rubber dam (IA1); latex-free rubber dam made of elastic plastomer (IA2), nitrile gloves (IA3); latex gloves (IA4); liquid rubber dam (IA5); 0.1 mm Teflon (IA6) and AZ strips (IA7). For investigating the HF permeability, each isolation agent to be tested was positioned over pH indicator paper and sealed airtight with a liquid rubber dam (OpalDam, Ultradent Products). The HF was then applied to each isolation agent. The pH value was determined on the pH indicator paper with the color scale chart of the manufacturer after 5 and 30 min. The duration of HF exposure to the isolation agents was set to 5 min and 30 min to simulate the duration of a minor intraoral repair and a more extensive repair or cementation, assuming that hydrofluoric acid was spilled and not completely removed from these isolation agents during this process.

**Table 1:** Overview of tested isolation agents

Material	Manufacturer	Abbreviation
Latex rubber dam Ivory rubber dam	Kulzer; Hanau, Germany	IA1
Elastic plastomer rubber dam Roeko flexi dam non latex	Coltene Holding; Altstätten, Switzerland	IA2
Nitrile gloves Nitra-tex micro touch	Ansell; Richmond, Australia	IA3
Latex gloves Dermagrip examination gloves	Remesco; Wien, Austria	IA4
Liquid rubber dam OpalDam	Ultradent Products; South Jordan, UT, USA	IA5
Teflon	W. Kirchhoff; Wallenhorst, Germany	IA6
AZ strip	frasaco; Tettnang, Germany	IA7

### Investigation of Neutralizing Agents

To neutralize HF four neutralizing agents (n = 8) were tested: NA1: calcium carbonate (Carl Roth; Karlsruhe, Germany) plus chlorhexidine gel (chlorhexidine digluconate 10 mg/g gel, GlaxoSmithKline Consumer Healthcare; Munich, Germany); NA2: calcium hydroxide (Calxyl, OCO Präparate; Dirmstein, Germany); NA3: calcium D-gluconate monohydrate (Sigma-Aldrich; St. Louis, Missouri, USA) plus chlorhexidine gel (chlorhexidine digluconate 10 mg/g gel; GlaxoSmithKline Consumer Healthcare); NA4: IPS neutralizing powder (Ivoclar; Schaan, Liechtenstein) plus water. For clinical intraoral application, a viscous or gel-like consistency of the neutralizing agent is favorable; chlorhexidine gel was therefore added to some agents.

The neutralization of HF was based on the following calculation shown in Table 2. All neutralizing agents were used in excess.

**Table 2:** Details of calculation of the neutralization of HF for each neutralizing agent

Group	Neutralizing agent (NA)	NA required for neutralization of 1 g (0.05 mol)/100 g solution HF^3^)	Theoretical weight ratio for neutralization	Used weight ratio^4^)
HF+NA1	CaCO_3_ (98.5%)^1^), M = 100.09 g/mol	2.5 g (0.025 mol)/22.5 g	1:0.23	1:0.5
HF+NA2	Ca(OH)_2_ (23.0%), M = 74.09 g/mol	1.85 g (0.025 mol)/16.7 g	1:0.73	1:1.5
HF+NA3	CaC_12_H_22_O_14_ · H_2_O (98.0%)^1^), M = 448.39 g/mol	11.2 g (0.025 mol)/100.9 g	1:1.03	1:2
HF+NA4	IPS neutralizing powder^2^): Na_2_CO_3_ ≥ 25% CaCO_3_ ≥ 25% M = 105.99/100.09 g/mol	Na_2_CO_3_: 2.65 g (0.025 mol)/23.85 g CaCO_3_: 2.5 g (0.025 mol)/22. 5 g	1:0.46	1:1

1) Addition of chlorhexidine gel until a paste was formed 2) Addition of water until a paste was formed 3) HF solution (9 %) 4) All neutralizing agents were used in excess

The pH value of each neutralizing agent was determined with pH indicator paper in wet conditions before and after mixing HF and the neutralizing agent (n = 8) with a microbrush (Young Innovations Europe; Heidelberg, Germany) on a rubber dam, followed by a 30-s reaction phase.

### Investigation of the Surface of Lithium Disilicate Ceramic

**Fig 2a-d:**
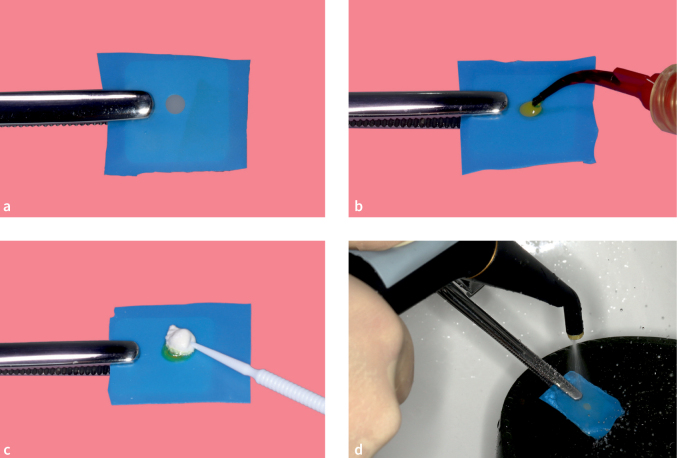
Etching and neutralization process of lithium disilicate ceramic, including (a) positioning rubber dam over specimen; (b) applying HF; (c) neutralizing the HF; and (d) rinsing with water spray after the reaction phase.

To investigate the surface of lithium disilicate ceramic after the etching and neutralizing process, lithium disilicate ceramic blocks (IPS e.max CAD, Ivoclar) were cut into disks with a thickness of 2 mm using a precision saw (Isomet 1000, Buehler; Leinfelden-Echterdingen, Germany) with water cooling. All specimens were crystalized according to the instructions of the manufacturer. The specimens were wet polished with 600- and 1200-grit abrasive silica carbide paper (CarbiMet; Buehler) in a grinding machine (Ecomet250 pro, Buehler). The specimens were cleaned in an ultrasonic bath in demineralized water followed by 99% isopropanol for 3 min. Forty specimens were divided into five groups (n = 8): HF: etching with 9% HF (served as the control group); HF+NA1: etching with 9% HF and neutralizing with calcium carbonate plus chlorhexidine gel; HF plus NA2: etching with 9% HF and neutralizing with calcium hydroxide; HF+NA3: etching with 9% HF and neutralizing with calcium D-gluconate monohydrate plus chlorhexidine gel; HF+NA4: etching with 9% HF and neutralizing with IPS neutralizing powder plus water. A new latex rubber dam with a hole (3 mm in diameter) was positioned over each specimen, to minimize and standardize the specimens’ surface area to be etched to adhere to the exact application times (Fig 2a). The application time for HF was set to 20 s for each group (Fig 2b).^11^ The neutralizing agent of the respective group was applied in the in excess calculated ratio (Table 3) and mixed with the HF using a micro brush (except group HF), followed by a 30-s reaction phase (Fig 2c). Then, the specimens were rinsed with water spray from a multifunction handpiece of a dental unit (KaVo Esthetica E70 Vision, KaVo Dental; Biberach an der Riß, Germany) for 30 s (Fig 2d) and dried with oil-free air.

**Table 3:** Batch number of materials used

Material	Manufacturer	Batch no.
Latex rubber dam Ivory rubber dam	Kulzer; Hanau, Germany	DL04APS2-BEM6
Elastic plastomer rubber dam Roeko flexi dam non latex	Coltene Holding; Altstätten, Switzerland	J167329
Nitrile gloves Nitra-tex micro touch	Ansell; Richmond, Australia	22108M2BDF
Latex gloves Dermagrip examination gloves	Remesco; Wien, Austria	104756202
Liquid rubber dam OpalDam	Ultradent Products; South Jordan, UT, USA	BPBSM
AZ strips	Frasaco; Tettnang, Germany	33550
Lithium disilicate ceramic IPS e.max CAD	Ivoclar; Schaan, Liechtenstein	S27004
9% Hydrofluoric acid Porcelain Etch	Ultradent Porcelain Etch, Ultradent Products; South Jordan, UT, USA	BPSYT
Chlorhexidine digluconate 10 mg/g gel	GlaxoSmithKline Consumer Healthcare; Munich, Germany	5152869
Calcium carbonate	Carl Roth; Karlsruhe, Germany	123328186
Calcium hydroxide Calxyl	OCO Präparate, Dirmstein, Germany	230302
IPS neutralizing powder	Ivoclar; Schaan, Liechtenstein	Z056C4

### SEM and EDX Analysis

Each specimen was sputter coated with a 10 nm gold layer (Leica EM QSG 100, Germany). The surface of each lithium disilicate ceramic specimen was examined for precipitates using scanning electron microscopy (SEM) at different magnifications up to 10,000×. When precipitates were detected energy-dispersive X-ray (EDX) analysis was performed to determine any changes in their elemental compositions. The data were obtained by an SEM (Zeiss Supra 55V) fitted with an EDX spectrometer (Oxford Xmax50). The primary electron energy used varied from 5 to 15 keV.

### Statistical Analysis

The collected data was coded, tabulated, and evaluated with a software program (Microsoft Excel, Redmond, WA, US).

## Results

### Preliminary Tests

A pH value of 2 was determined for the 9% HF for intraoral use. The preliminary test with the watch glass also led to a pH value of 2 within 60 s without the indicator paper coming into direct contact with the HF, which indicates the evaporation of HF. The pilot study of the HF permeability of isolation agents was consistent with the results of the main study.

### Investigation of Isolation Agents

**Fig 3:**
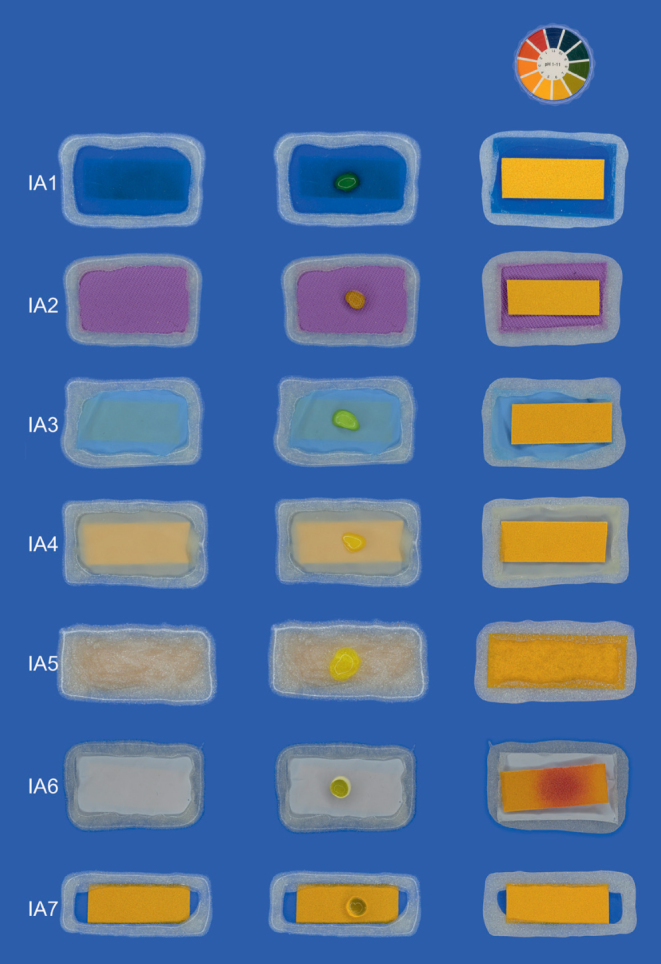
Permeability of isolation agents. Column 1: IA1–IA7 sealed airtight with liquid rubber dam over pH indicator paper. Column 2: Application of HF to IA1-IA7. Column 3: Back side of IA1-IA7 showing the indicator paper after 30 min.

When investigating the isolation agents with airtight conditions after HF exposure for 5 min and 30 min, no change in pH value was determined, except for group IA6 (Fig 3). A pH value of 2 was determined after 5 and 30 min for every specimen of this group, proving that Teflon, unlike the other isolation agents, was permeable to HF.

### Investigation of Neutralizing Agents

**Fig 4:**
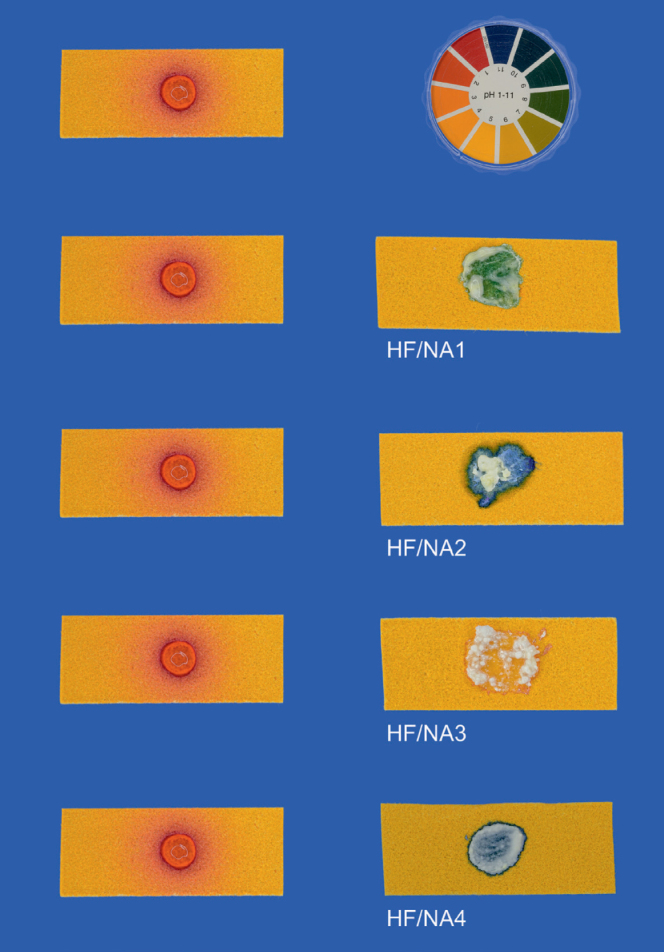
pH value after neutralizing HF with NA1-NA4. Column 1: pH indicator paper with HF prior to the neutralization process. Column 2: pH indicator paper after neutralizing HF with NA1–NA4.

Mean pH values of neutralizing agents before and after neutralizing HF are illustrated in Table 4 and Figure 4. Within each test group all eight specimens showed the same pH value before the neutralization process. After the neutralization process, the same pH value was also observed for all eight specimens within each test group with the exception of HF+NA1.

**Table 4:** Mean and standard deviation (SD) of pH values for each neutralizing agent

Neutralizing agent (NA)	Abbreviation	Mean pH value before neutralization	Mean pH value ± SD after neutralization
Calcium carbonate plus chlorhexidine gel	NA1	9	8.9 ± 0.3
Calcium hydroxide	NA2	11	11
Calcium D-gluconate monohydrate plus chlorhexidine gel	NA3	8	6
IPS neutralizing powder plus water	NA4	11	11

When determining the pH value after neutralization, the pH indicator paper showed a lower pH value in the peripheral area than in the center of the applied mixture of NA and HF due to diffusion or evaporation of the not completely neutralized HF. This effect was observed in group HF+NA3 for each of the eight specimens. Visually the mixture was framed in the color of lower pH (pH = 2), as shown in Figure 4.

Mean central pH values after neutralizing HF ranged from a minimum of 6 for group HF+NA3 to a maximum of 11 for groups HF+NA2 and HF+NA4. The pH values confirmed, that HF could be neutralized with all tested neutralizing agents used in the in excess calculated ratio under laboratory conditions.

### Investigation of the Surface of Lithium Disilicate Ceramic

**Fig 5a-e:**
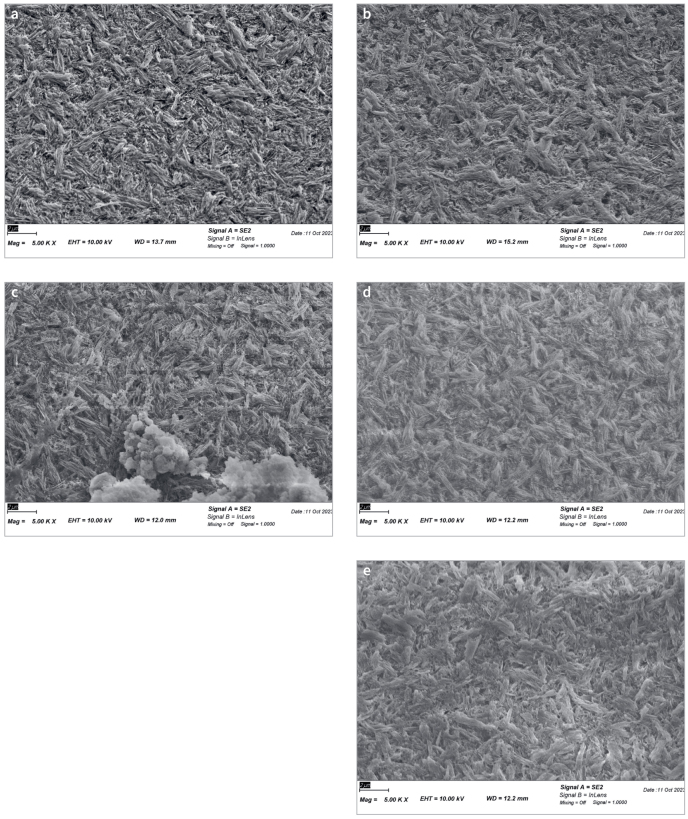
Exemplary SEM images of lithium disilicate ceramic surface with different surface treatments: *(a)* HF; *(b)* HF+NA1;* (c)* HF+NA2; *(d)* HF+NA3; and *(e)* HF+NA4.

Each specimen was investigated with SEM. Assessment with SEM showed precipitates on two surfaces of lithium disilicate ceramic specimens in group HF+NA2 only (Fig 5c). The surface of both specimens showed precipitates scattered individually. For all other neutralization groups, no precipitates were found on the specimens’ surfaces, and the SEM images were comparable with the images of the control group HF, hydrofluoric acid etching without neutralization (Fig 5a, 5b, 5d, 5e).

For group HF+NA2, EDX analysis confirmed residues of calcium fluoride, among others such as barium (Table 5). EDX analysis showed peaks at 0.87 keV for fluoride and 3.66 keV for calcium.

**Table 5:** Atomic percentage of elements of an exemplary etched ceramic surface with and without precipitates (HF+NA3)

Element	Atomic % of a	
	surface with no precipitates	Surface with precipitates
O	65.47	59.46
C	8.41	12.63
Si	21.58	19.22
Na	0.35	0.44
K	1.75	1.48
Au	1.9	1.52
Al	0.54	–
F	–	2.77
Ca	–	1.55
Ba	–	0.58
S	–	0.35

## Discussion

Although HF is available for intraoral use, eg, for intraoral repair of glass-ceramic restorations, the research is limited regarding its use, neutralization, and adequate isolation methods. This laboratory study aimed to investigate the permeability to HF of common materials, various neutralizing agents, and their effect on the surface of lithium disilicate ceramics.

The preliminary test of this study confirmed that HF evaporates, which is consistent with the safety data sheet and the instructions of the manufacturer (Ultradent).^37^ Studies have reported about the risks of vapor and inhalation of hydrogen fluoride.^3,6,25^ Therefore, it is even more important to create airtight conditions and use a vapor tip during the intraoral application of HF.

The first null hypothesis was rejected for Teflon but accepted for all other materials which were impermeable to HF at 30 min, and therefore seem to be suitable and mandatory for clinical use. When using HF intraorally, the manufacturer (Ultradent) claims that a rubber dam or Etch Arrest, a sodium bicarbonate and calcium source acid neutralization medium, or Opal dam must be used to protect nearby tissue and restorations.^37^ Despite the fact that the neutralizing agent Etch Arrest is no longer available on the European market, the neutralizing agent alone would not prevent the vapor of HF and protect it from inhalation or ingestion. Further, Teflon is recommended to protect adjacent tissue and restorations, also in a step-by-step video of the manufacturer on “How to use Ultradent Porcelain Repair Kit.”^17,37^ Those recommendations are not consistent with the findings of this study, even though Teflon has been described not to be affected by reagents such as hydrogen fluoride.^10^

The second hypothesis was accepted as all tested neutralizing agents were able to neutralize the HF under laboratory conditions. All neutralizing agents reached their original pH value except for the agent calcium D-gluconate-monohydrate (98%) plus chlorhexidine gel. An explanation for this might be that the mixing of this agent with HF was not sufficient due to its granular consistency, as the powder did not dissolve completely. This could also explain the fact that the pH values were lower at the edges of the mixture in this group.

The IPS neutralization powder has also been used in other studies, which concluded that this agent decreased the resin bond strength to ceramic.^2,5,28^ Amaral et al observed precipitates on the ceramic surface after etching with a 5% HF for 1 min followed by application of this neutralizing agent, washing and drying, and sonic cleaning in distilled water for 5 min.^2^ This, however, is not in agreement with the results of group HF+NA4 in this study. However, the findings of the aforementioned study can only be compared with the present study to a limited extent, as the studies differ in the used ceramics, including different structures and compositions (feldspar vs lithium disilicate ceramic), etching times (60 s vs 20 s), HF concentration (5% vs 9%), and in the neutralization (powder versus powder mixed with water) and cleaning processes (ultrasonic cleaning versus water rinsing). Additionally, precipitates were found on feldspar ceramic surfaces after etching with 9% HF without neutralization. As no EDX analysis was performed, it is questionable whether the precipitates were caused by the neutralizing agent or by the acid conditioning followed by insufficient cleaning. A negative effect of ultrasonic cleaning in distilled water for zirconia ceramic has been observed previously showing a decrease of the adhesion efficacy to a resin-luting material.^23^ However, it is unclear whether this also applies to feldspar ceramic. Another study used calcium gluconate with water, and also calcium hydroxide and calcium carbonate, but in powder form and without additional components such as chlorhexidine gel or water as used in the present study. SEM analysis revealed no debris or acid-etched residues. SEM images of the groups with neutralizing agents showed a similar morphology to that of the group in which only HF etching was performed.^29^ This was partially consistent with the results of the current study, as no precipitates were observed on the surfaces, with the exception of group HF+NA2. Additionally, the SEM images were comparable with the images of the control group HF. While the aforementioned study showed no fluoride element on any of the specimens (with a peak at 0.67 (Kα) keV) during EDX analysis,^29^ the EDX analysis of the current study confirmed residues of calcium fluoride with peaks at 0.87 keV for fluoride and 3.66 keV for calcium, among other residues of the ingredients of Calxyl, as barium, in group HF+NA2 (Table 5). An explanation for the precipitates found in this group might be due to the shorter rinsing time of 30 s compared with the rinsing time of 60 s of the aforementioned study. However, the comparison is limited, as the neutralization process was not described in detail in the aforementioned studies, no mixing ratio was specified, and no pH values were measured. Mostly, only powders have been used for neutralizing. With regard to intraoral application, the use of powder alone seems unsuitable. Rather, the neutralizing agent’s viscous or gel-like consistency is of interest, enabling the neutralizing agent to frame the surface to be etched and serve as a barrier to protect the remaining restoration. Except for group HF+NA3, all neutralizing agents showed a suitable consistency.

Limitations of this study included measuring the pH value with indicator paper, which is less objective and less precise than a pH meter. However, a quantitative pH measurement was not required in this study as the pH values were only to confirm the neutralization of each agent, because the neutralization was apparent from the calculations and was used in excess. Another limitation of this study was that no resin bond strength test was performed after the etching and neutralization process simulating an intraoral repair. Therefore, possible clinical behavior is less predictable. Further, the selected neutralizing agents are not approved for the indication used in this study.

Currently, no neutralizing agent is approved for the intraoral use of HF when repairing glass-ceramic restorations, which means that clinicians are faced with the dilemma of using products with neutralizing properties as an off-label use, skipping the neutralization process with an increasing risk of HF exposure due to water spray or inadequate suctioning, or not using hydrofluoric acid at all. As the use of glass-ceramic restorations is increasing, ceramic repairs remain necessary, and etching with hydrofluoric acid is still the gold standard, further research is required.

## Conclusion

The HF for intraoral use evaporated, making the use of a vapor tip and isolation agents essential to protect patients and clinicians during intraoral ceramic repair.

All tested isolation agents, with the exception of Teflon, were impermeable to hydrofluoric acid at 30 min when sealed airtight with a flowable rubber dam, and therefore, seem suitable for clinical use.

HF could be neutralized with all tested neutralizing agents used in the excess calculated ratio under laboratory conditions. However, sufficient mixing with NA3 was difficult due to its consistency, so it does not seem suitable for clinical use. When using NA2 for the neutralization of HF on a lithium disilicate ceramic, precipitates could remain on the surface, which might influence the resin bond strength. Therefore, an extension of the rinsing time needs to be considered.
